# External validation of an opioid misuse machine learning classifier in hospitalized adult patients

**DOI:** 10.1186/s13722-021-00229-7

**Published:** 2021-03-17

**Authors:** Majid Afshar, Brihat Sharma, Sameer Bhalla, Hale M. Thompson, Dmitriy Dligach, Randy A. Boley, Ekta Kishen, Alan Simmons, Kathryn Perticone, Niranjan S. Karnik

**Affiliations:** 1 Division of Health Informatics and Data Science, Loyola University Chicago, Maywood, IL USA; 2 Department of Medicine, University of Wisconsin, 1685 Highland Avenue, Madison, WI 53705 USA; 3 Department of Psychiatry and Behavioral Sciences, Rush University Medical Center, Chicago, IL USA; 4 Rush Medical College, Rush University, Chicago, IL USA; 5 Department of Computer Science, Loyola University Chicago, Chicago, IL USA; 6 Clinical Research Analytics, Research Core, Rush University Medical Center, Chicago, IL USA

**Keywords:** Opioid misuse, Heroin, Opioid use disorder, Natural language processing, Machine learning, Computable phenotype

## Abstract

**Background:**

Opioid misuse screening in hospitals is resource-intensive and rarely done. Many hospitalized patients are never offered opioid treatment. An automated approach leveraging routinely captured electronic health record (EHR) data may be easier for hospitals to institute. We previously derived and internally validated an opioid classifier in a separate hospital setting. The aim is to externally validate our previously published and open-source machine-learning classifier at a different hospital for identifying cases of opioid misuse.

**Methods:**

An observational cohort of 56,227 adult hospitalizations was examined between October 2017 and December 2019 during a hospital-wide substance use screening program with manual screening. Manually completed Drug Abuse Screening Test served as the reference standard to validate a convolutional neural network (CNN) classifier with coded word embedding features from the clinical notes of the EHR. The opioid classifier utilized all notes in the EHR and sensitivity analysis was also performed on the first 24 h of notes. Calibration was performed to account for the lower prevalence than in the original cohort.

**Results:**

Manual screening for substance misuse was completed in 67.8% (n = 56,227) with 1.1% (n = 628) identified with opioid misuse. The data for external validation included 2,482,900 notes with 67,969 unique clinical concept features. The opioid classifier had an AUC of 0.99 (95% CI 0.99–0.99) across the encounter and 0.98 (95% CI 0.98–0.99) using only the first 24 h of notes. In the calibrated classifier, the sensitivity and positive predictive value were 0.81 (95% CI 0.77–0.84) and 0.72 (95% CI 0.68–0.75). For the first 24 h, they were 0.75 (95% CI 0.71–0.78) and 0.61 (95% CI 0.57–0.64).

**Conclusions:**

Our opioid misuse classifier had good discrimination during external validation. Our model may provide a comprehensive and automated approach to opioid misuse identification that augments current workflows and overcomes manual screening barriers.

**Supplementary Information:**

The online version contains supplementary material available at 10.1186/s13722-021-00229-7.

## Background

Opioid-related inpatient and emergency department (ED) visits have increased 64% since 2009, and the rate of opioid-related ED visits has nearly doubled through 2014, including recent rises during the COVID-19 pandemic [1, 2]. Many patients engage the health system for the first time after a physical health complication related to opioid misuse such as endocarditis or respiratory infection [3]. The care team is frequently focused on the acute physical ailment and not the patient’s opioid misuse. Large treatment gaps continue to exist as hospitals serve a high concentration of individuals with opioid misuse who do not receive screening, especially when admitted for another condition [4]. Existing universal screening questionnaires such as the interviewer-administered screening questions [5] require significant staff time and training to administer. Further, patients may be reluctant to report stigmatized behavior to an interviewer [6, 7]. Overall, conventional screening methods are resource-intensive and face significant barriers to successful implementation in a hospital setting [8].

Routinely collected data in the electronic health record (EHR) may be leveraged to identify cases of opioid misuse. Patients are more likely to disclose substance use to their hospital primary care team than to designated screeners who are not part of the care team [9, 10]. The admission notes and social history sections of notes written by the provider teams frequently contain details about substance use but are rarely accessed for surveillance or screening programs. Computational methods of natural language processing (NLP) can derive discrete representations of clinical notes, from which machine learning can predict opioid misuse better than rule-based approaches [11–14].

We previously published and made publicly available an opioid misuse classifier using NLP and machine learning from the clinical notes [15]. In hospitalized patients, our convolutional neural network (CNN) outperformed a rule-based approach and other machine learning methods. Our CNN opioid classifier had 79% sensitivity and 91% specificity, and our results showed that clinical notes from the hospitalization can be used to identify opioid misuse and serve as an alternative to manual screening by staff. Our opioid classifier was originally trained and calibrated in a source cohort of high-risk inpatients at Loyola University Medical Center. The trained model comprised of 15,651 medical concepts from 63,301 notes fed into the CNN [15]. The top positive features included terms such as ‘heroin’, ‘opiates’, ‘drug abuse’, and ‘polysubstance abuse’. However, the CNN is a non-linear model with many potential interactions and combination of concepts so external validation is vital prior to deployment. The previously developed CUI-based opioid classifier is accessible at https://github.com/AfsharJoyceInfoLab/OpioidNLP_Classifier.

We aim to externally validate our opioid classifier against manual screening in an independent health system (Rush University Medical Center) that instituted hospital-wide screening for all hospital admissions since 2017. We hypothesized that our opioid classifier would provide sensitivity and specificity above 80%.

## Methods

### Source of data and participants

Rush University Medical Center (Rush) is a 727-bed hospital, tertiary care academic center serving Chicago with Epic (Epic Systems Corporation, Verona, Wisconsin) as its EHR vendor. Rush launched a multidisciplinary Substance Use Intervention Team (SUIT) to address the opioid epidemic through a Screening, Brief Intervention, and Referral to Treatment (SBIRT) program with an inpatient Addiction Consult Service in October 2017 [16]. Part of the SUIT program included the following single question universal drug screen: “How many times in the past year have you used an illegal drug or used a prescription medication for non-medical reasons?” (> 1 is positive). The single-question screen was administered by nursing staff to patients admitted to Rush’s 18 inpatient medical and surgical wards. Patients with a positive universal screen were referred for a full screen with the 10-item Drug Abuse Screening Test (DAST-10) [17].

The inclusion criteria were all unplanned adult inpatient encounters (≥ 18 years of age) who were screened between October 23, 2017 and December 31, 2019. Unplanned admissions were defined using the Center for Medicare and Medicaid Services (CMS) rules for unplanned admission [18]. Outpatient encounters or discharges from the ED were excluded and patients that did not receive a universal screen and/or DAST-10 were excluded. The original development and internal validation cohort for training the NLP classifier was from Loyola University Medical Center using a sampling of hospitalized patients with an over-sampling of individuals with risk factors for opioid misuse [15].

### Reference standard: outcome for testing opioid misuse classifier

Reference cases of opioid misuse for testing against the machine learning algorithm were determined using the DAST-10 [19]. We used a cutoff score of ≥ 2 for a positive screen for any substance misuse, which has been shown to have favorable sensitivity and specificity in identifying substance misuse in healthcare settings [17]. The type of substance use, including opioid misuse, was also collected in patients with a positive DAST-10. Opioid misuse was defined as patients with a DAST ≥ 2 and taking an opioid for reasons other than prescribed or as an illicit drug [20]. The final labels of positive cases in the reference cohort included patients with opioid misuse, either alone or in combination with other drugs.

### Predictors from clinical notes

The manual screen data (e.g., questionnaire data) collected by hospital staff into EHR flowsheets were excluded from the extraction of predictors (i.e., features) to avoid any contamination of the reference data in the test dataset. Linguistic processing to extract all features from the clinical notes (i.e., admission note, progress note, consult note, ancillary notes, etc.) was performed using the clinical Text Analysis and Knowledge Extraction System (cTAKES; http://ctakes.apache.org) [21]. cTAKES can recognize words or phrases from text as medical terms and maps them to the National Library of Medicine’s Unified Medical Language System (UMLS), which includes over 2 million clinical concepts merged into the National Library of Medicine Metathesaurus. The spans of the UMLS Metathesaurus named entity mentions (diseases, symptoms, anatomical sites, drugs, and procedures) were mapped from the Rush EHR clinical notes and organized into Concept Unique Identifiers (CUIs), which are structured codes derived from multiple medical vocabularies. For instance, *‘heroin abuse’* is assigned C0600241 as its CUI which also includes eight other synonyms. ‘*Heroin abuse’* is mapped to a separate CUI than ‘*history of heroin abuse*’ which is C3266350. The classifier was fed all the CUI predictors/features as inputs into a CUI embedding that was analyzed by our previously trained convolutional neural network (CNN).

### Error analysis on misclassifications between the automated NLP opioid misuse classifier and the self-report manual screen

Post-hoc chart review was performed in cases where the NLP classifier was deemed a false-positive or false-negative against the manual screen. A trained annotator (SB) performed chart review to provide a final likelihood for opioid misuse using all the data available in the EHR. The annotator met an inter-rater reliability of > 0.80 with an addiction specialist (KP) before independent review was performed. The final likelihood for opioid misuse included a Likert scale for definite, highly probable, probable, definitely not, and uncertain for determining opioid misuse. These criteria were developed by consensus using a Delphi approach between a board-certified clinical informatics specialist and internist, board-certified addiction medicine specialist, and board-certified psychiatrist [15]. Substance use characteristics and treatments were compared across Likert groups and displayed in Table 3.

Probable cases required any one of the following: (1) history of opioid misuse evident in the clinical notes but no current documentation for the encounter; (2) provider mention of aberrant drug behavior; (3) evidence of other drug misuse (except alcohol) in addition to prescription opioid use; (4) documented history of opioid misuse but in remission, thus remaining at-risk. Highly probable cases were classified by more than one of the probable case criteria, or provider mention of opioid dependence plus suspicion of misuse in the clinical notes. Definite cases were classified as the patient self-reporting opioid misuse to a provider or documentation by provider of patient currently misusing an opioid. The remainder of cases were categorized as no opioid misuse.

### Analysis plan

Statistical tests to compare baseline patient characteristics between opioid misuse and no misuse groups were conducted using the chi-square test for proportions and Wilcoxon-Mann Whitney nonparametric tests for integer variables. Comorbid conditions were defined with International Classification of Disease (ICD) codes based on the Elixhauser comorbidity categories [22]. Missing data analysis was performed to compare the manually screened hospitalizations to the hospitalized group that did not received screening (Additional file 1: Table S1).

The primary outcome was discrimination of the opioid classifier for identifying opioid misuse versus no opioid misuse as measured by the Area Under the Receive Operating Characteristic Curve (AUROC) and the Precision-Recall Curve (PR Curve). The PR Curve is a better discrimination metric for unbalanced datasets [23]. The following test characteristics and their 95% confidence intervals (CI) were reported: sensitivity, specificity, negative predictive value (NPV), and positive predictive value (PPV). An optimal cut-point level was derived by examining a range of cutpoints including Youden indice [24]. Sensitivity analysis was performed by running the classifier using only the first 24 h of notes to better reflect its potential use as an inpatient screening tool.

The adjustment of models across settings with different prevalence rates, so-called model updating or recalibration, is recommended by the Transparent Reporting of a Multivariable Prediction Model for Individual Prognosis or Diagnosis (TRIPOD) guidelines [25] to avoid over- or underestimation of a patient’s risk. We anticipated these issues may occur in external validation and provided results for both uncalibrated and calibrated models. Calibration plots were examined to assess the reliability and agreement of the classifier predictions against the reference standard. Calibration was formally assessed by the calibration slope, intercept, and visually with a calibration plot. A non-parametric regression with isotonic calibration was used to account for the decrease in prevalence. Isotonic calibration provides a piecewise linear model to predict the sequences of observations that preserves the order as a monotonic function for uncalibrated estimates from our model [26].

Analysis was performed using Python Version 3.6.5 (Python Software Foundation) and RStudio Version 1.1.463 (RStudio Team, Boston, MA). The Institutional Review Board of Rush approved this study. We followed the 2015 guideline for Transparent Reporting of a multivariable Prediction Model for Individual Prognosis Or Diagnosis (TRIPOD): Prediction Model Validation Checklist (Additional file 1: Table S3).

## Results

**Fig. 1:**
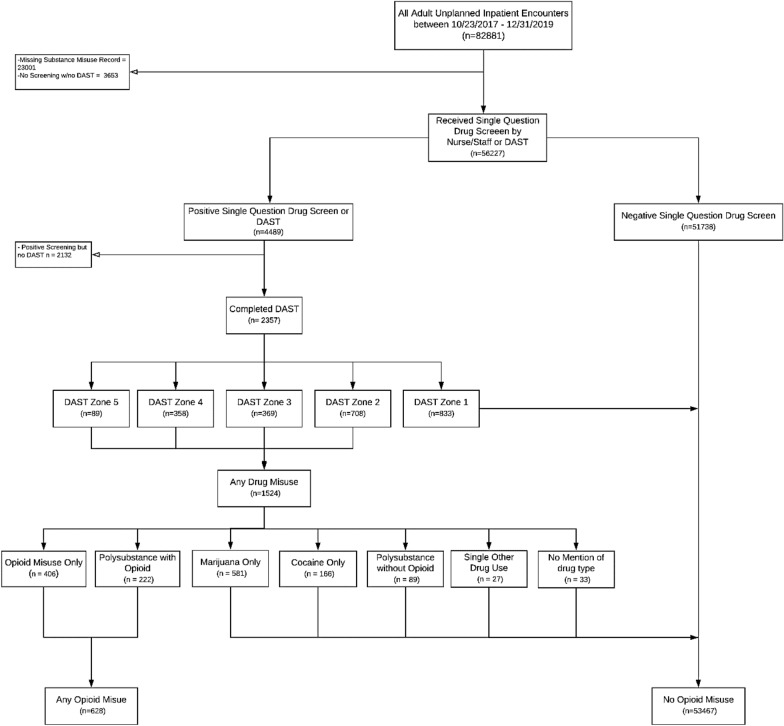
Patient hospital encounter flow chart during study period. Unplanned admissions were defined using the Center for Medicare and Medicaid Services. Single question screen was performed by the nurse with the following: ““How many times in the past year have you used an illegal drug or used a prescription medication for non-medical reasons? (> 1 is positive)”. DAST = drug abuse screening tool; Zone 1 = DAST score 0; Zone 2 = DAST score 1; Zone 3 = DAST score 2–5; Zone 4 = DAST score 6–8; Zone 5 = DAST score 9–10

During the study period, there were 82,881 unplanned adult hospitalizations and DAST screening for substance misuse was completed in 67.8% (n = 56,227) with 1.1% (n = 628) of the screened cohort identified with opioid misuse (Fig. 1). The cohort that did not have screening data recorded in the EHR was similar in demographics to the cohort with screening data (Additional file 1: Table S1). Comparisons were made between self-report opioid misuse (via questionnaire) and no misuse (Table 1). A lower proportion of co-morbidities and substance misuse by ICD codes and a higher proportion of in-hospital death was found in the group without opioid misuse versus the group with opioid misuse by manual screen. The median age of patients with opioid misuse was younger than without misuse, and a greater proportion of patients with opioid misuse were male and non-Hispanic black (p < 0.01) (Table 1). A greater proportion also had chronic lung disease, depression, polysubstance drug use, and psychiatric conditions (p < 0.01). More patients with opioid misuse were on Medicaid and were discharged against medical advice than patients without misuse (Table 1).

**Table 1:** Baseline patient characteristics and outcomes between self-report opioid misuse (via questionnaire) and no misuse

Characteristics and outcomes	Opioid misuse (n = 628)	No misuse (n = 53467)	p-value
Age, median (IQR)	49 (38–8)	61 (45–71)	< 0.001
Male Sex, n (%)	400 (63.6%)	22055 (41.2%)	< 0.001
Race/Ethnicity, n (%)			< 0.001
Non-Hispanic White	196 (31.2%)	23221 (43.4%)	
Non-Hispanic Black	336 (53.5%)	17232 (32.2%)	
Hispanic White	26 (4.1%)	2927 (5.5%)	
Hispanic Black	2 (< 1%)	134 (< 1%)	
Other	68 (10.8%)	9953 (18.6%)	
DAST Score (median, IQR, n = 16,453)	6 (4–8)	0 (0–1)	< 0.001
Insurance, n (%)			< 0.001
Medicare	79 (12.5%)	20074 (37.5%)	
Medicaid	65 (74.0%)	18136 (33.9%)	
Private	84 (13.4%)	14686 (27.4%)	
Other	0 (0%)	571 (1.1%)	
Elixhauser Comorbidities, n (%)			
Hypertension, uncomplicated	163 (25.9%)	17686 (33.1%)	< 0.001
Hypertension, complicated	150 (23.8%)	14970 (27.9%)	0.025
Diabetes Mellitus, uncomplicated	25 (3.9%)	3718 (6.9%)	0.005
Diabetes Mellitus, complicated	83 (13.2%)	11277 (21.1%)	< 0.001
Renal Failure	93 (14.8%)	11045 (20.7%)	< 0.001
Neurologic Disorders	100 (15.9%)	8471 (15.8%)	0.999
Congestive Heart Failure	117 (18.6%)	9670 (18.1%)	0.764
Liver Disease	94 (14.9%)	3772 (7.1%)	< 0.001
Chronic Lung Disease	236 (37.5%)	10633 (19.9%)	< 0.001
Psychoses	119 (18.9%)	2072 (3.8%)	< 0.001
Depression	146 (23.2%)	7974 (14.9%)	< 0.001
Alcohol Abuse	116 (18.4%)	1957 (3.6%)	< 0.001
Drug Abuse	605 (96.3%)	1190 (2.2%)	< 0.001
AIDS/HIV	22 (3.5%)	389 (< 1%)	< 0.001
Discharge Disposition, n (%)			< 0.001
Home	355 (56.5%)	31224 (57.1%)	
In-Hospital Death	3 (< 1%)	590 (1.1%)	
Long or Shorter Term Care	120 (19.1%)	7150 (13.4%)	
Against Medical Advice	60 (9.5%)	365 (< 1%)	
Other	90 (14.3%)	14138 (26.4%)	

**Fig. 2:**
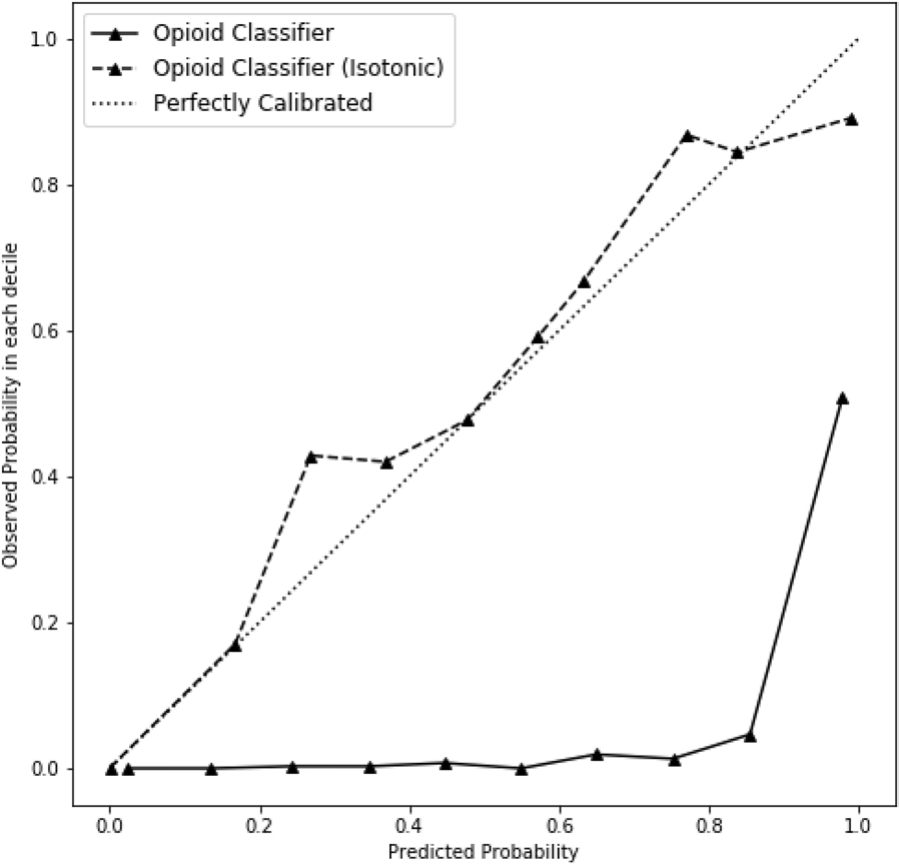
Calibration plots for uncalibrated and calibrated opioid misuse classifier. Calibration compares observed and predicted probabilities across deciles of predicted probabilities. Uncalibrated classifier is solid line and isotonic calibration is dashed line

For external validation of the opioid classifier, the 56,227 hospital encounters included 2,482,900 clinical notes and 67,969 unique CUIs. The opioid misuse classifier had an AUROC of 0.99 (95% CI 0.99–0.99), and a PR AUC of 0.79 (95% CI 0.75–0.82). The optimal cutpoint had a sensitivity and specificity of 0.98 (95% CI 0.98–0.98) and 0.98 (95% CI 0.98–0.98), respectively. The corresponding PPV and NPV were 0.37 (95% CI 0.35–0.39) and 0.99 (95% CI 0.99–0.99). Calibration plot for the uncalibrated model demonstrates over-prediction across all deciles of predicted probabilities; therefore, the model was calibrated for the lower prevalence in our hospitalized cohort and provided a better model fit (Fig. 2). In the calibrated model, the calibration slope was 1.10 (95% CI 1.05–1.88) and calibration intercept was −5.09 (95% CI −5.40 to −4.81). In the calibrated model, the optimal cutpoint had a sensitivity and specificity of 0.81 (95% CI 0.77−0.84) and 0.99 (95% CI 0.99−0.99). The corresponding PPV and NPV were 0.72 (95% CI 0.68−0.75) and 0.99 (95% CI 0.99−0.99), respectively. This would create approximately 2 alerts per day for every 100 patients, and one in every 1.4 alerts would be a true positive (number needed to evaluate of 1.4). A range of cutpoints are shown in Table 2.

**Table 2:** Test characteristics of Opioid Classifier across a range of cutpoints in calibrated model

Cutpoint	Sensitivity (95% CI)	Specificity (95% CI)	PPV (95% CI)	NPV (95% CI)
0.35	0.86 (0.83, 0.89)	0.99 (0.99, 0.99)	0.67 (0.64, 0.71)	0.99 (0.99, 0.99)
0.40	0.83 (0.80, 0.86)	0.99 (0.99, 0.99)	0.69 (0.65, 0.72)	0.99 (0.99, 0.99)
0.42^a^	0.81 (0.77, 0.84)	0.99 (0.99, 0.99)	0.7 (0.67, 0.74)	0.99 (0.99, 0.99)
0.45	0.79 (0.75, 0.82)	0.99 (0.99, 0.99)	0.71 (0.67, 0.74)	0.99 (0.99, 0.99)
0.50^a^	0.78 (0.74, 0.81)	0.99 (0.99, 0.99)	0.72 (0.68, 0.75)	0.99 (0.99, 0.99)
0.55	0.64 (0.60, 0.68)	0.99 (0.99, 0.99)	0.77 (0.74, 0.81)	0.99 (0.99, 0.99)
0.60	0.56 (0.52, 0.60)	0.99 (0.99, 0.99)	0.82 (0.78, 0.85)	0.99 (0.99, 0.998)

^a^Youden’s (*J*) Statistic; PPV, positive predictive value; NPV,  negative predictive value

In sensitivity analysis, the opioid classifier was tested on the first 24 h of clinical notes after arrival to the hospital (Additional file 1: Table S2). The data included 649,419 clinical notes with 54,763 unique CUIs. The opioid classifier had an AUROC of 0.98 (95% CI 0.98–0.99), and a PR AUC of 0.71 (95% CI 0.67–0.75). In the calibrated model, the calibration slope was 0.99 (95% CI 0.95–1.03) and calibration intercept was − 4.17 (95% CI − 4.33 to – 4.01). The optimal cutpoint had a sensitivity and specificity of 0.75 (95% CI: 0.71–0.78) and 0.99 (95% CI: 0.99–0.99), respectively. The corresponding PPV and NPV were 0.61 (95% CI 0.57–0.64) and 0.99 (95% CI 0.99–0.99). This would create approximately 2 alerts per day for every 100 patients, and one in every 1.6 alerts would be a true positive (number needed to evaluate of 1.6).

Error analysis by chart review of the uncalibrated model identified 1.9% (n = 1091) misclassifications between the opioid classifier and manual screening. In 99% (n = 1081) of the discordant cases between the NLP classifier and the self-report manual screen reference standard, the NLP classifier labelled cases as positive but the self-report manual screens were negative. However, 49.3% (n = 533) of these cases were noted to have at least a probable likelihood for opioid misuse after post-hoc chart review by the annotator, suggesting the NLP classifier correctly labelled the cases as positive and under-reporting occurred during the self-report manual screen (Table 3). Of the cases with at least a probable likelihood, 64.7% (n = 345) were determined by the reviewer to be true positives because of prior evidence for misuse in the EHR notes. As the likelihood for opioid misuse increased on the Likert scale by the chart reviews, the predicted probability of the opioid classifier increased as well (Table 3).

**Table 3:** Characteristics of misclassifications (false-positives and false-negatives) between opioid classifier and manual screen (n = 1091)

Patient characteristics	Likelihood of opioid misuse in patient chart review				p value
	Definitely	Highly Probable	Probable	Definitely Not	
n	99	21	413	558	
Predicted probability from opioid classifier (mean ± SD)	0.96 ± 0.07	0.93 ± 0.11	0.88 ± 0.12	0.81 ± 0.12	< 0.001
Age (mean ± SD)	49.9 ± 13.3	52.4 ± 13.8	53.3 ± 12.3	45.3 ± 14.4	< 0.001
Chronic pain, n (%)	26 (26.3%)	6 (28.6%)	127 (30.8%)	107 (19.2%)	0.001
Prior mention of opioid misuse, n (%)	92 (92.9%)	18 (85.7%)	263 (63.7%)	2 (0.4%)	< 0.001
Trauma or intoxication evidence, n (%)	12 (12.1%)	0 (0%)	5 (1.2%)	2 (0.4%)	< 0.001
Withdrawal or overdose symptoms, n (%)	14 (14.1%)	1 (4.8%)	15 (3.6%)	5 (0.9%)	< 0.001
Naloxone administered, n (%)	2 (2%)	0 (0%)	5 (1.2%)	7 (1.3%)	0.869
Positive drug screen, n (%)					
Opiates	38 (38.4%)	9 (42.9%)	74 (17.9%)	56 (10.0%)	< 0.001
Benzodiazepines	11 (11.1%)	3 (14.3%)	37 (9.0%)	104 (18.6%)	< 0.001
Cannabinoids	11 (11.1%)	3 (14.3%)	28 (6.8%)	78 (14.0%)	0.005
Cocaine metabolites	26 (26.3%)	2 (9.5%)	45 (10.9%)	73 (13.1%)	0.001
Other	3 (3%)	2 (9.5%)	14 (3.4%)	35 (6.3%)	0.116
All negative	25 (25.3%)	5 (23.8)	70 (16.9%)	162 (29.0%)	< 0.001
Not available	38 (38.4%)	10 (47.6%)	254 (61.5%)	164 (29.4%)	< 0.001
Administered opioids during encounter, n (%)	37 (37.4%)	9 (42.9%)	163 (39.5%)	117 (21.0%)	< 0.001
Administered benzodiazepines during encounter, n (%)	17 (17.2%)	3 (14.3%)	37 (9.0%)	210 (37.6%)	< 0.001
Opioids prescribed upon discharge, n (%)	15 (15.2%)	3 (14.3%)	83 (20.1%)	58 (10.4%)	< 0.001
Other substance misuse, n (%)	56 (56.6%)	13 (61.9%)	239 (57.9%)	373 (66.8%)	0.021
Alcohol	21	5	84	256	< 0.001
Cocaine metabolites	39	6	166	132	< 0.001
Cannabis	17	5	78	103	0.91
Other	1	2	30	27	0.079
Diagnosed psychiatric disorders, n (%)					
Depression	22 (22.2%)	2 (9.5%)	101 (24.5%)	198 (35.5%)	< 0.001
Anxiety	15 (15.2%)	2 (9.5%)	78 (18.9%)	127 (22.8%)	0.126
Bipolar	14 (14.1%)	5 (23.8%)	36 (8.7%)	82 (14.7%)	0.015
Schizophrenia	5 (5.1%)	0 (0%)	8 (1.9%)	23 (4.1%)	0.154
Post traumatic disorder	2 (2.0%)	2 (9.5%)	18 (4.4%)	40 (7.2%)	0.082
Other	9 (9.1%)	1 (4.8%)	22 (5.3%)	45 (8.1%)	0.315
No psychiatric history	55 (55.6%)	14 (66.7%)	244 (59.1%)	240 (43.0%)	< 0.001

## Discussion

Our opioid misuse classifier had good discrimination and calibration in external validation in a cohort of hospitalized patients, and it provided a sensitivity and specificity above 95% using the full encounter of notes. Limiting the data to the first 24 h of the hospital encounter, which accounted for 25% of the clinical notes, led to a small drop in performance but continued to demonstrate a sensitivity and specificity at 75% or above. Error analysis revealed that under-reporting is common with many of the false-positives being deemed as true-positives based on chart review. Our model may provide a comprehensive and automated approach to opioid misuse identification that may augment current workflow and potentially overcome the current manual screening rate of 68%.

Currently, single questionnaire screens for drugs represent universal screening tools supported by national practice guidelines [19, 27]. Published results demonstrate 82% sensitivity and 74% specificity for illicit or nonmedical prescription drug misuse [5]. Our opioid misuse classifier achieved similar results given a large available clinical narrative, including sensitivity analysis with the first 24 h of notes. In 2016, there were about 35.7 million hospital stays with a mean length of stay of 4.6 days [28]—ample time for the classifier to also be used for screening and providing an intervention after the first 24 h of the encounter.

The United States Preventive Task Force emphasizes screening tools that do not include drug testing [19, 29, 30]. The USPSTF conducted a systematic review and identified 30 different screening tools, often with a sensitivity of more than 75% for detecting substance misuse, and report that most studies used structured clinical or diagnostic interview [31]. Post-hoc chart review of the misclassifications by the NLP opioid misuse classifier against the manual self-report questionnaire data showed nearly half of the misclassifications of false-positives by the NLP classifier were re-labelled as true positives after in-depth chart review across the patient’s hospital encounter. The discrepancy between self-report and clinical documentation possibly reflects underreporting to the screener or missing information not captured in the structured interviews but available in the provider notes. This highlights the value of notes for additional information that may not be captured in self-report.

Poor screening and treatment options have led to less than a quarter of patients with opioid misuse receiving treatment—suggesting we need better approaches to identify and treat patients [32, 33]. A systematic review on automatable algorithms for opioid misuse revealed the data used in many published algorithms are not routinely available in the EHR, or some algorithms rely solely on diagnostic billing codes which have poor sensitivity [34]. To date, best performing algorithms depend on pharmacy claims data which are not available in EHRs; therefore, impractical to providers and hospitals [35–37]. There is little direct evidence to demonstrate the application of NLP and machine learning in routinely collected EHR notes to identify patients with opioid misuse. Validation of our opioid misuse classifier enables a standardized approach to perform screening on patient encounters. This study is a step toward a more automated screening tool that can potentially overcome the current screening rate of 68%. In addition, the NLP classifier may identify additional cases missed by the DAST, which accounted for another 533 positive cases during post-hoc chart review. Because the tool is derived from notes collected during routine care, it may also benefit health systems that do not have mature screening programs with customized data entry.

The descriptive statistics about our cohort support the classifier’s performance from the notes. Many of the patients identified by our opioid classifier also had ICD codes for drug misuse with high rates of mental health conditions and alcohol use disorders which are risk factors associated with opioid misuse [35, 38, 39]. In our chart review of over 1000 encounters, those with a higher likelihood for opioid misuse by the human reviewer also had, on average, a greater predicted probability for opioid misuse by the classifier.

The prevalence of opioid misuse in our health system of 1.1% was similar to other reports in hospitalized patients [40, 41]. A national study from the National Emergency Department Sample with data from over 234 million adult ED visits found 1.23% of all visits were related to opioid-related diagnoses (opioid use, dependence, withdrawal, and other related conditions with opioid use such as mental and behavioral disorders associated with opioid use) [42]. In our original development study for the opioid classifier [15], we derived a source cohort with approximately a third having opioid misuse which led to an over prediction by our classifier when applied to Rush University Hospital which has a lower case-rate. Calibration is frequently under-reported for published models but a major reason for failure in models to perform well in external settings and to capture appropriate risk among groups [25, 43]. As the severity of the opioid crisis varies over time and by region, prediction performance may also change across hospitals and the populations they serve. This phenomenon is well-described and has been labelled as calibration drift [25]. Continually training a new model is not feasible because it is time-consuming, requires abundant data, and wastes potentially useful information from existing models [44]. Therefore, updating trained models is the appropriate alternative and we demonstrate that calibration of our model to account for changes in case-rates and setting improved the PPV from approximately 37% to 71%. The higher PPV confers a lower number needed to evaluate and is more effective at limiting false alerts to reduce alarm fatigue.

Several limitations for application of the opioid misuse classifier exist. There remains a paucity of evidence into the benefits and harms of screening so the role for automated algorithms to improve health outcomes remains unclear. Quasi-experimental designs like an interrupted time-series to evaluate the replacement of current practice automated tools are next steps in evaluating the effectiveness of the NLP classifier. We provide credence in the predictive and face validity of the tool, but prospective designs are needed for casual inference on health outcomes. Current experiments for deployment of the opioid misuse classifier are registered in clinicaltrials.gov (NCT03833804). Outcomes such as receipt of motivational interviewing, initiation of buprenorphine, and re-hospitalization are among the outcomes of interest. Further, the capital costs for informatics teams at health systems to process clinical notes at point-of-care are substantial [45] and cost analyses are needed to evaluate the resource allocation needs for machine learning algorithms.

Our health system’s screening system with the DAST was among the recommended instruments by the National Institute of Drug Abuse (NIDA) [46]. However, it is not the gold standard and has been largely evaluated in psychiatric outpatient settings with little data on its predictive validity in hospitalized patients [17]. Other instruments have reported similar or better sensitivity, including the Tobacco, Alcohol, Prescription medications, and other Substance (TAPS) tool or the World Health Organization World Mental Health Composite International Diagnostic Interview [46, 47]. Although our tool identified over 500 potential cases not detected by the manual screen, there were also approximately another 500 cases that were confirmed to be false-positives. Mislabeling individuals with opioid misuse can be highly stigmatizing and additional bias and equity assessments are needed prior to deployment. Treatment may vary across individuals with different levels of misuse, such as unhealthy use versus substance use disorders, but our data did not allow such differentiation to be analyzed. Clinical trials are needed to examine the benefit of machine learning algorithms over existing screening methods and their efficacy in hospitalized patients.

In conclusion, in external validation of our opioid classifier, we demonstrate high accuracy after calibration for identifying hospitalized patients with opioid misuse. An automated NLP algorithm using routinely gathered EHR data may help health systems provide comprehensive screening for targeted interventions.

## Supplementary Information

**Additional file 1: Table S1.** Characteristics between patients with and without screening data. **Table S2.** Test characteristics on first 24 h of notes for opioid classifier (uncalibrated and calibrated for 24 h). **Table S3.** TRIPOD Checklist: Prediction Model Validation.

## Data Availability

The trained models generated from this study are available on the corresponding author’s GitHub repository (https://github.com/AfsharJoyceInfoLab/OpioidNLP_Classifier). The datasets used and/or analyzed during the current study are available from Rush University Chicago after a data use agreement and IRB approval are established between Rush University Medical Center and the interested party.

## Abbreviations

PHI: Protected health information
CUI: Concept unique identifier
NLP: Natural language processing
EHR: Electronic health record
ICD: International classification of disease
cTAKES: Clinical text and knowledge extraction system
UMLS: Unified medical language system
ROC AUC: Receiver operating characteristic area under the curve
PPV: Positive predictive value
NPV: Negative predictive value
CNN: Convolutional neural network

## References

[1] Weiss AJ, Elixhauser A, Barrett ML, Steiner CA, Bailey MK and O’Malley L. Opioid-related inpatient stays and emergency department visits by state, 2009–2014: statistical brief# 219. 2017. 28682575

[2] McCann Pineo M, Schwartz RM (2020). Commentary on the coronavirus pandemic: anticipating a fourth wave in the opioid epidemic. Psychol Trauma.

[3] Weiss AJ, Bailey MK, O’Malley L, Barrett ML, Elixhauser A, Steiner CA (2017). Patient characteristics of opioid-related inpatient stays and emergency department visits nationally and by state, 2014: statistical brief# 224.

[4] Naeger S, Mutter R, Ali MM, Mark T, Hughey L (2016). Post-discharge treatment engagement among patients with an opioid-use disorder. J Subst Abuse Treat.

[5] Smith PC, Schmidt SM, Allensworth-Davies D, Saitz R (2010). A single-question screening test for drug use in primary care. Arch Intern Med.

[6] Bradley KA, Lapham GT, Hawkins EJ, Achtmeyer CE, Williams EC, Thomas RM, Kivlahan DR (2011). Quality concerns with routine alcohol screening in VA clinical settings. J Gen Intern Med.

[7] Wright RG, Rotheram-Borus MJ, Klosinski L, Ramos B (2000). Screening for transmission behaviors among HIV-infected adults. AIDS Educ Prev..

[8] Barbosa C, Cowell AJ, Landwehr J, Dowd W, Bray JW (2016). Cost of screening, brief intervention, and referral to treatment in health care settings. J Subst Abuse Treat.

[9] Smothers BA, Yahr HT (2005). Alcohol use disorder and illicit drug use in admissions to general hospitals in the United States. Am J Addict.

[10] Boniface S, Kneale J, Shelton N (2014). Drinking pattern is more strongly associated with under-reporting of alcohol consumption than socio-demographic factors: evidence from a mixed-methods study. BMC Public Health.

[11] Gonzalez-Hernandez G, Sarker A, O’Connor K, Savova G (2017). Capturing the patient’s perspective: a review of advances in natural language processing of health-related text. Yearb Med Inform.

[12] Dligach D, Afshar M, Miller T (2019). Toward a clinical text encoder: pretraining for clinical natural language processing with applications to substance misuse. J Am Med Inform Assoc.

[13] Afshar M, Phillips A, Karnik N, Mueller J, To D, Gonzalez R, Price R, Cooper R, Joyce C, Dligach D (2019). Natural language processing and machine learning to identify alcohol misuse from the electronic health record in trauma patients: development and internal validation. J Am Med Inform Assoc.

[14] To D, Sharma B, Karnik N, Joyce C, Dligach D, Afshar M (2020). Validation of an alcohol misuse classifier in hospitalized patients. Alcohol.

[15] Sharma B, Dligach D, Swope K, Salisbury-Afshar E, Karnik NS, Joyce C, Afshar M (2020). Publicly available machine learning models for identifying opioid misuse from the clinical notes of hospitalized patients. BMC Med Inform Decision Making..

[16] Thompson HM, Hill K, Jadhav R, Webb TA, Pollack M, Karnik N (2019). The substance use intervention team: a preliminary analysis of a population-level strategy to address the opioid crisis at an academic health center. J Addict Med.

[17] Yudko E, Lozhkina O, Fouts A (2007). A comprehensive review of the psychometric properties of the Drug Abuse Screening Test. J Subst Abuse Treat.

[18] Desai NR, Ross JS, Kwon JY (2016). Association between hospital penalty status under the hospital readmission reduction program and readmission rates for target and nontarget conditions. JAMA.

[19] Force USPST, Krist AH, Davidson KW, Mangione CM, Barry MJ, Cabana M, Caughey AB, Curry SJ, Donahue K, Doubeni CA, Epling JW, Kubik M, Ogedegbe G, Pbert L, Silverstein M, Simon MA, Tseng C-W, Wong JB (2020). Screening for unhealthy drug use: US preventive services task force recommendation statement. JAMA..

[20] Quality USDoH, Human Services Substance A, Mental Health Services Administration Center For Behavioral Health Statistics A, United States Department of H, Human Services. Substance A, Mental Health Services Administration. Center for Behavioral Health S and Quality. National Survey on Drug Use and Health, 2011. ICPSR Data Holdings. 2012.

[21] Savova GK, Masanz JJ, Ogren PV, Zheng J, Sohn S, Kipper-Schuler KC, Chute CG (2010). Mayo clinical Text Analysis and Knowledge Extraction System (cTAKES): architecture, component evaluation and applications. J Am Med Inform Assoc.

[22] Elixhauser A, Steiner C, Harris DR, Coffey RM (1998). Comorbidity measures for use with administrative data. Med Care.

[23] Saito T, Rehmsmeier M (2015). The precision-recall plot is more informative than the ROC plot when evaluating binary classifiers on imbalanced datasets. PLoS ONE.

[24] Schisterman EF, Perkins NJ, Liu A, Bondell H (2005). Optimal cut-point and its corresponding Youden Index to discriminate individuals using pooled blood samples. Epidemiology.

[25] Moons KGM, Altman DG, Reitsma JB, Ioannidis JPA, Macaskill P, Steyerberg EW, Vickers AJ, Ransohoff DF, Collins GS (2015). Transparent reporting of a multivariable prediction model for Individual Prognosis or Diagnosis (TRIPOD): explanation and elaboration. Ann Intern Med.

[26] Huang Y, Li W, Macheret F, Gabriel RA, Ohno-Machado L (2020). A tutorial on calibration measurements and calibration models for clinical prediction models. J Am Med Inform Assoc.

[27] Dowell D, Haegerich TM, Chou R (2016). CDC guideline for prescribing opioids for chronic pain—United States, 2016. JAMA.

[28] Weiss AJ, Elixhauser A. Overview of Hospital Stays in the United States, 2012: Statistical Brief #180 *Healthcare Cost and Utilization Project (HCUP) Statistical Briefs* Rockville (MD): Agency for Healthcare Research and Quality (US); 2014. 25506966

[29] Feldman Y, Koren G, Mattice D, Shear H, Pellegrini E, MacLeod SM (1989). Determinants of recall and recall bias in studying drug and chemical exposure in pregnancy. Teratology.

[30] Garg M, Garrison L, Leeman L, Hamidovic A, Borrego M, Rayburn WF, Bakhireva L (2016). Validity of self-reported drug use information among pregnant women. Matern Child Health J.

[31] Patnode CD, Perdue LA, Rushkin M, Dana T, Blazina I, Bougatsos C, Grusing S, O'Connor EA, Fu R, Chou R (2020). Screening for unhealthy drug use: updated evidence report and systematic review for the us preventive services task force. JAMA.

[32] Trowbridge P, Weinstein ZM, Kerensky T, Roy P, Regan D, Samet JH, Walley AY (2017). Addiction consultation services–linking hospitalized patients to outpatient addiction treatment. J Subst Abuse Treat.

[33] Grant BF, Saha TD, June Ruan W, Goldstein RB, Patricia Chou S, Jung J, Zhang H, Smith SM, Pickering RP, Huang B, Hasin DS (2016). Epidemiology ofDSM-5drug use disorder. JAMA Psychiatry.

[34] Canan C, Polinski JM, Alexander GC, Kowal MK, Brennan TA, Shrank WH (2017). Automatable algorithms to identify nonmedical opioid use using electronic data: a systematic review. J Am Med Inform Assoc.

[35] Hylan TR, Von Korff M, Saunders K, Masters E, Palmer RE, Carrell D, Cronkite D, Mardekian J, Gross D (2015). Automated prediction of risk for problem opioid use in a primary care setting. J Pain.

[36] Smith RC, Frank C, Gardiner JC, Lamerato L, Rost KM (2010). Pilot study of a preliminary criterion standard for prescription opioid misuse. Am J Addict.

[37] Lo-Ciganic W-H, Huang JL, Zhang HH, Weiss JC, Wu Y, Kent Kwoh C, Donohue JM, Cochran G, Gordon AJ, Malone DC, Kuza CC, Gellad WF (2019). Evaluation of machine-learning algorithms for predicting opioid overdose risk among medicare beneficiaries with opioid prescriptions. JAMA Network Open.

[38] Sullivan MD, Edlund MJ, Zhang L, Unützer J, Wells KB (2006). Association between mental health disorders, problem drug use, and regular prescription opioid use. Arch Intern Med.

[39] Edlund MJ, Steffick D, Hudson T, Harris KM, Sullivan M (2007). Risk factors for clinically recognized opioid abuse and dependence among veterans using opioids for chronic non-cancer pain. Pain.

[40] McNeely J, Gourevitch MN, Paone D, Shah S, Wright S, Heller D (2012). Estimating the prevalence of illicit opioid use in New York City using multiple data sources. BMC Public Health.

[41] Kassed CA, Levit KR and Hambrick MM. Hospitalizations Related to Drug Abuse, 2005: Statistical Brief #39 Healthcare Cost and Utilization Project (HCUP) Statistical Briefs Rockville (MD): Agency for Healthcare Research and Quality (US); 2007. 21850770

[42] Langabeer JR, Stotts AL, Bobrow BJ (2021). Prevalence and charges of opioid-related visits to U.S. emergency departments. Drug Alcohol Depend..

[43] Collins GS, de Groot JA, Dutton S, Omar O, Shanyinde M, Tajar A, Voysey M, Wharton R, Yu L-M, Moons KG, Altman DG (2014). External validation of multivariable prediction models: a systematic review of methodological conduct and reporting. BMC Med Res Methodol.

[44] Siregar S, Nieboer D, Versteegh MIM, Steyerberg EW, Takkenberg JJM (2019). Methods for updating a risk prediction model for cardiac surgery: a statistical primer. Interact Cardiovasc Thorac Surg.

[45] Afshar M, Dligach D, Sharma B, Cai X, Boyda J, Birch S, Valdez D, Zelisko S, Joyce C, Modave F (2019). Development and application of a high throughput natural language processing architecture to convert all clinical documents in a clinical data warehouse into standardized medical vocabularies. J Am Med Inform Assoc..

[46] National Institute on Drug Abuse (June 22, 2018). Screening and Assessment Tools Chart. https://www.drugabuse.gov/nidamed-medical-health-professionals/screening-tools-resources/chart-screening-tools. Accessed 8 Feb 2021

[47] Kessler RC, Usun BT (2004). The World Mental Health (WMH) Survey Initiative version of the World Health Organization (WHO) Composite International Diagnostic Interview (CIDI). Int J Methods Psychiatr Res.

