# Supplementary material for: External validation of an opioid misuse machine learning classifier in hospitalized adult patients
Source: Addict Sci Clin Pract. 2021 Mar 17;16:19. doi: 10.1186/s13722-021-00229-7 (PMC7967783; doi:10.1186/s13722-021-00229-7)
Supplement: Supplementary file 1 — Additional file 1: Table S1. Characteristics between patients with and without screening data. Table S2. Test characteristics on first 24 h of notes for opioid classifier (uncalibrated and calibrated for 24 h). Table S3. TRIPOD Checklist: Prediction Model Validation. [file 13722_2021_229_MOESM1_ESM.docx]

**Online Supplement: External Validation of an Opioid Misuse Machine Learning Classifier in Hospitalized Adult Patients**

Majid Afshar, MD, MSCR^1^; Brihat Sharma, MS^2^; Sameer Bhalla, BS^3^; Hale M. Thompson, PhD^2^; Dmitriy Dligach, PhD^4^; Randy A. Boley, BA^2^; Ekta Kishen, MPH^5^; Alan Simmons, BS^5^; Jawad Khan, MS^6^; Kathryn Perticone, APN, MSW^2^; Niranjan S. Karnik, MD, PhD^2^

^1^ Department of Medicine, University of Wisconsin, Madison, WI

^2^ Department of Psychiatry & Behavioral Sciences, Rush University Medical Center, Chicago, IL

^3^ Rush Medical College, Rush University, Chicago, IL

^4^ Department of Computer Science, Loyola University Chicago, Chicago, IL

^5^ Clinical Research Analytics, Research Core, Rush University Medical Center, Chicago, IL

^6^ Knowledge Management, Rush University Medical Center, Chicago, IL

Corresponding Author:

Majid Afshar, MD, MSCR

Department of Medicine

1685 Highland Avenue

Madison, WI 53705

Phone: +1-608-263-1792

Email: [majid.afshar@wisc.edu](mailto:majid.afshar@wisc.edu)

**Table S1.** Characteristics between patients with and without screening data

|  | Analytic Cohort (54095) | Missing Cohort  (28786) | p-value |
| --- | --- | --- | --- |
| Age, median (IQR) | 61 (47 - 71) | 62 (46 - 73) | <0.001 |
| Male Sex, n (%) | 22455 (41.5%) | 12299 (42.7%) | <0.001 |
| Race/Ethnicity, n (%)  Non-Hispanic White  Non-Hispanic Black  Hispanic White  Hispanic Black  Other | 23417 (43.3%) 17568 (32.5%) 2953 (5.5%) 136 (<1%) 10021 (18.5%) | 12583 (43.7%) 10157 (35.2%) 1247 (4.3%) 60 (<1%) 4739 (16.4%) | <0.001 |
| Insurance, n(%)  Medicare Medicaid Private Other | 20153 (37.3%) 18601 (34.4%)  14770 (27.3%)  571 (1.1%) | 9295 (32.3%) 11352 (39.4%)  7909 (27.4%)  230 (<1%) | <0.001 |
| Discharge Disposition(n%) Home: In-Hosital Death: LT RC / ST PA: AMA: Other: | 31579 (58.3%)  595 (1.1%)  7270 (13.4%) 425 (<1%) 14226 (26.3%) | 15133 (52.5%) 1779 (6.2%) 3632 (12.6%) 356 (1.2%) 7886 (27.4%) | <0.001 |
| HTN, n (%) | 17849 (32.9%) | 7617 (26.5%) | <0.001 |
| TUMOR, n (%) | 3913 (7.2%) | 1328 (4.6%) | <0.001 |
| RENLFAIL, n (%) | 11138 (20.6%) | 5444 (18.9%) | <0.001 |
| OBESE, n (%) | 18896 (34.9%) | 7885 (27.4%) | <0.001 |
| NEURO, n (%) | 8571 (15.8%) | 4553 (15.8%) | 0.925 |
| CHF, n (%) | 9787 (18.1%) | 4851 (16.9%) | <0.001 |
| DMCX, n (%) | 11360 (21.0%) | 5135 (17.8%) | <0.001 |
| LYTES, n (%) | 15790 (29.1%) | 7614 (26.5%) | <0.001 |
| WGHTLOSS, n(%) | 10035 (18.5%) | 4090 (14.2%) | <0.001 |
| LIVER, n(%) | 3866 (7.1%) | 1388 (4.8%) | <0.001 |
| CHRNLUNG, n(%) | 10869 (20.1%) | 5436 (18.8%) | <0.001 |
| VALVE, n(%) | 4147 (7.7%) | 1689 (5.9%) | <0.001 |
| METS, n(%) | 3310 (6.1%) | 1312 (4.6%) | <0.001 |
| ANEMDEF, n(%) | 15674 (28.9%) | 6180 (21.4%) | <0.001 |
| PERIVASC, n(%) | 3950 (7.3%) | 1563 (5.4%) | <0.001 |
| DM, n(%) | 3743 (6.9%) | 1932 (6.7%) | 0.265 |
| LYMPH, n(%) | 1299 (2.4%) | 386 (1.3%) | <0.001 |
| PSYCH, n(%) | 2191 (4.1%) | 1885 (6.5%) | <0.001 |
| DEPRESS, n(%) | 8120 (15.0%) | 3695 (12.8%) | <0.001 |
| COAG, n(%) | 4548 (8.4%) | 1795 (6.2%) | <0.001 |
| PULMCIRC, n(%) | 1448 (2.7%) | 610 (2.1%) | <0.001 |
| ARTH, n(%) | 2647 (4.9%) | 969 (3.3%) | <0.001 |
| HTNCX, n(%) | 15120 (27.9%) | 7465 (25.9%) | <0.001 |
| PARA, n(%) | 3573 (6.6%) | 1694 (5.9%) | <0.001 |
| ALCOHOL, n(%) | 2073 (3.8%) | 1344 (4.7%) | <0.001 |
| DRUG, n(%) | 1795 (3.3%) | 1507 (5.2%) | <0.001 |
| HYPOTHY, n(%) | 6694 (12.4%) | 2895 (10.1%) | <0.001 |
| AIDS, n(%) | 411 (<1%) | 231 (<1%) | 0.531 |
| BLDLOSS, n(%) | 1799 (3.3%) | 1066 (3.7%) | 0.004 |
| ULCER. n(%) | 721 (1.3%) | 314 (1.1%) | 0.003 |

**Table S2.** Test characteristics on first 24 hour of notes for opioid classifier (uncalibrated and calibrated for 24 hour)

Uncalibrated

| Cutpoint | Sensitivity (95% CI) | Specificity (95% CI) | PPV (95% CI) | NPV (95% CI) |
| --- | --- | --- | --- | --- |
| 0.30 | 0.97(0.95, 0.98) | 0.94(0.94, 0.94) | 0.16(0.15, 0.18) | 0.99(0.99, 0.99) |
| 0.35 | 0.96 (0.94,0.97) | 0.96 (0.96,0.96) | 0.23 (0.21, 0.25) | 0.99(0.99, 0.99) |
| 0.40 | 0.95 (0.93, 0.96) | 0.97 (0.97, 0.97) | 0.28 (0.26, 0.30) | 0.99(0.99, 0.99) |
| 0.45* | 0.94 (0.92, 0.96) | 0.98 (0.97, 0.98) | 0.31 (0.29, 0.34) | 0.99(0.99, 0.99) |
| 0.50 | 0.94 (0.91, 0.95) | 0.98 (0.98, 0.98) | 0.35 (0.33, 0.37) | 0.99(0.99, 0.99) |
| 0.52* | 0.97(0.95, 0.98) | 0.94(0.94, 0.94) | 0.16(0.15, 0.18) | 0.99(0.99, 0.99) |
| 0.55 | 0.96 (0.94,0.97) | 0.96 (0.96,0.96) | 0.23 (0.21, 0.25) | 0.99 (0.99, 0.99) |

Calibrated

| Cutpoint | Sensitivity (95% CI) | Specificity (95% CI) | PPV (95% CI) | NPV (95% CI) |
| --- | --- | --- | --- | --- |
| 0.30 | 0.75 (0.71, 0.78) | 0.99(0.99, 0.99) | 0.61(0.57, 0.64) | 0.99(0.99, 0.99) |
| 0.35 | 0.70 (0.66, 0.73) | 0.99(0.99, 0.99) | 0.64 (0.60, 0.68) | 0.99(0.99, 0.99) |
| 0.40 | 0.62 (0.58, 0.66) | 0.99(0.99, 0.99) | 0.70 (0.66, 0.74) | 0.99(0.99, 0.99) |
| 0.45* | 0.59 (0.55, 0.62) | 0.99(0.99, 0.99) | 0.73 (0.69, 0.77) | 0.99(0.99, 0.99) |
| 0.50 | 0.55 (0.51, 0.59) | 0.99(0.99, 0.99) | 0.75 (0.71, 0.79) | 0.99(0.99, 0.99) |
| 0.52* | 0.52 (0.48, 0.56) | 0.99(0.99, 0.99) | 0.78(0.73, 0.82) | 0.99(0.99, 0.99) |
| 0.55 | 0.51 (0.47, 0.55) | 0.99 (0.99, 0.99) | 0.77 (0.73, 0.81) | 0.99 (0.99, 0.99) |

*Youden’s (*J*) Statistic

**Table S3. TRIPOD Checklist: Prediction Model Validation**

| **Section/Topic** | **Item** | **Checklist Item** | **Page** |
| --- | --- | --- | --- |
| **Title and abstract** | | | |
| Title | 1 | Identify the study as developing and/or validating a multivariable prediction model, the target population, and the outcome to be predicted. | **1** |
| Abstract | 2 | Provide a summary of objectives, study design, setting, participants, sample size, predictors, outcome, statistical analysis, results, and conclusions. | **2-3** |
| **Introduction** | | | |
| Background and objectives | 3a | Explain the medical context (including whether diagnostic or prognostic) and rationale for developing or validating the multivariable prediction model, including references to existing models. | **3-4** |
|  | 3b | Specify the objectives, including whether the study describes the development or validation of the model or both. | **3-4** |
| **Methods** | | | |
| Source of data | 4a | Describe the study design or source of data (e.g., randomized trial, cohort, or registry data), separately for the development and validation data sets, if applicable. | **4-5** |
|  | 4b | Specify the key study dates, including start of accrual; end of accrual; and, if applicable, end of follow-up. | **4-5** |
| Participants | 5a | Specify key elements of the study setting (e.g., primary care, secondary care, general population) including number and location of centres. | **4-5** |
|  | 5b | Describe eligibility criteria for participants. | **4-5** |
|  | 5c | Give details of treatments received, if relevant. | **4-5** |
| Outcome | 6a | Clearly define the outcome that is predicted by the prediction model, including how and when assessed. | **5-6** |
|  | 6b | Report any actions to blind assessment of the outcome to be predicted. | **5-6** |
| Predictors | 7a | Clearly define all predictors used in developing or validating the multivariable prediction model, including how and when they were measured. | **6-7** |
|  | 7b | Report any actions to blind assessment of predictors for the outcome and other predictors. | **6-7** |
| Sample size | 8 | Explain how the study size was arrived at. | **5** |
| Missing data | 9 | Describe how missing data were handled (e.g., complete-case analysis, single imputation, multiple imputation) with details of any imputation method. | **8** |
| Statistical analysis methods | 10c | For validation, describe how the predictions were calculated. | **7** |
|  | 10d | Specify all measures used to assess model performance and, if relevant, to compare multiple models. | **6-7** |
|  | 10e | Describe any model updating (e.g., recalibration) arising from the validation, if done. | **8** |
| Risk groups | 11 | Provide details on how risk groups were created, if done. | **7-8** |
| Development vs. validation | 12 | For validation, identify any differences from the development data in setting, eligibility criteria, outcome, and predictors. | **5** |
| **Results** | | | |
| Participants | 13a | Describe the flow of participants through the study, including the number of participants with and without the outcome and, if applicable, a summary of the follow-up time. A diagram may be helpful. | **Fig 1.** |
|  | 13b | Describe the characteristics of the participants (basic demographics, clinical features, available predictors), including the number of participants with missing data for predictors and outcome. | **10** |
|  | 13c | For validation, show a comparison with the development data of the distribution of important variables (demographics, predictors and outcome). | **Table 1** |
| Model performance | 16 | Report performance measures (with CIs) for the prediction model. | **12-13; Table 2** |
| Model-updating | 17 | If done, report the results from any model updating (i.e., model specification, model performance). | **12** |
| **Discussion** | | | |
| Limitations | 18 | Discuss any limitations of the study (such as nonrepresentative sample, few events per predictor, missing data). | **19-20** |
| Interpretation | 19a | For validation, discuss the results with reference to performance in the development data, and any other validation data. | **16-18** |
|  | 19b | Give an overall interpretation of the results, considering objectives, limitations, results from similar studies, and other relevant evidence. | **16-18** |
| Implications | 20 | Discuss the potential clinical use of the model and implications for future research. | **16-18** |
| **Other information** | | | |
| Supplementary information | 21 | Provide information about the availability of supplementary resources, such as study protocol, Web calculator, and data sets. | **Online supp** |
| Funding | 22 | Give the source of funding and the role of the funders for the present study. | **21-22** |
